# The ability of left ventricular end-diastolic volume variations measured by TEE to monitor fluid responsiveness in high-risk surgical patients during craniotomy: a prospective cohort study

**DOI:** 10.1186/s12871-017-0456-6

**Published:** 2017-12-04

**Authors:** Haidan Lan, Xiaoshuang Zhou, Jing Xue, Bin Liu, Guo Chen

**Affiliations:** Department of Anesthesiology, West China Hospital of Sichuan University, No.37, Guo Xue Xiang, Chengdu, 610041 Sichuan People’s Republic of China

**Keywords:** TEE, SVV, Left ventricular end-diastolic volume, Fluid responsiveness measurement

## Abstract

**Background:**

This study was aimed to evaluate the ability of left ventricular end-diastolic volume variations (LVEDVV) measured by transesophageal echocardiography (TEE) compared with stroke volume variation (SVV) obtained by the FloTrac/Vigileo monitor to predict fluid responsiveness, in patients undergoing craniotomy with goal direct therapy.

**Methods:**

We used SVV obtained by the FloTrac/Vigileo monitor to manage intraoperative hypotension in adult patients undergoing craniotomy (ASA III – IV) after obtaining IRB approval and informed consent. The LVEDVV were measured by TEE through the changes of left ventricular short diameter of axle simultaneously. When cardiac index (CI) ≤ 2.5 and SVV ≥ 15%, comparisons were made between the two devices before and after volume expansion.

**Results:**

We enrolled twenty-six patients referred for craniotomy in this study and 145 pairs of data were obtained. Mean Vigileo-SVV and TEE-LVEDVV were 17.8 ± 2.78% and 22.1 ± 7.25% before volume expansion respectively, and were 10.95 ± 2.8% and 13.58 ± 3.78% after volume expansion respectively (*P* < 0.001). The relationship between Vigileo-SVV and TEE-LVEDVV was significant (r2 = 0.55; *p* < 0.001). Agreement between Vigileo-SVV and TEE-LVEDVV was 3.3% ± 3.9% (mean bias ± SD, Bland-Altman).

**Conclusions:**

For fluid responsiveness of patients during craniotomy in ASA III-IV, LVEDVV measured by left ventricular short diameter of axle using M type echocaidiographic measurement seems an acceptable monitoring indicator. This accessible method has promising clinical applications in situations where volume and cardiac function monitoring is of great importance during surgery.

**Trial registration:**

Chinese Clinical Trial Registry, ChiCTR-TRC-13003583, August 20, 2013.

## Background

In high-risk patients undergoing craniotomy, accurate assessment of intravascular fluid status and measurement of fluid responsiveness is important since inadequate and excessive fluid replacement can affect postoperative clinical outcomes of high-risk patients [1–3]. Goal-directed therapy has been shown to be useful to improve the outcome of patients undergoing major surgery [4–6].

Stroke volume variation (SVV) is a reliably predictor of fluid loading response, which can be used to guide fluid therapy in mechanically ventilated patients [7–9]. A systematic meta-analysis demonstrated that SVV is useful to predict fluid responsiveness in many different settings [10], and could reliably predicts fluid responsiveness with an area under ROC curve of 0.8–0.9 [11, 12]. Transesophageal echocardiography (TEE) is used widely in the perioperative arena to monitor patients during cardiac and high-risk non-cardiac surgeries and life-threatening emergencies. It can provide qualitative and quantitative information on ventricular and valvular functions and dynamic cardiac monitoring [13]. Left ventricular end-diastolic volume (LVEDV) could be easily estimated by left ventricular short diameter of axle using M type echocardiographic measurement. Variation of LVEDV (LVEDVV) is a parameter to predict fluid responsiveness. However, the ability of LVEDVV to predict fluid responsiveness has not been fully evaluated.

We design this study to evaluate ability of LVEDVV measured by TEE to predict fluid responsiveness, and the relationship and the agreement between LVEDVV and SVV obtained by the FloTrac/Vigileo monitor in goal direct therapy of patients undergoing craniotomy.

## Methods

### Setting

This study was performed in Department of Anesthesiology at the West China Hospital of Sichuan University, Chengdu, China. This study was approved by the Ethical Committee of the West China Hospital from Sichuan University.

### Participants

After personal written informed consent were obtained, 26 adult patients undergoing elective craniotomy for brain tumor resection or intracranial aneurysm were considered for enrollment. Inclusion criteria were age > 18, ASA score III or IV, and expected duration of surgery >2 h. Patients were excluded for the study if the body weight < 40 kg or >100 kg, with significant cardiac arrhythmias, ventricular dysfunction, aortic aneurysm, extensive peripheral arterial occlusive disease, significant valvulopathy, intracardic shunt, ejection fraction <35%.

### Intraoperative monitoring and management

In addition to pulse oxymetry, capnography, and electrocardiograph monitoring, all patients had a radial arterial line in place for continuous blood pressure monitoring and BIS sensor in place to monitor the depth of anesthesia. Anesthesia induction was done with propofol (2 mg/kg), rocuronium (1 mg/kg) and sufentanil (0.5 μg/kg), then propofol and remifentanil were used to maintain depth of anesthesia (BIS in the range of 40–60 intraoperatively). Tidal volume was set at 8 ml/kg and respiratory frequency was adapted to maintain arterial partial pressure of end-tidal CO2 between 35 and 40 mmHg. The inspiratory to expiratory ratio was set to 1:2 with no PEEP (positive end expiratory pressure). According to our neurosurgical policy, all patients received mannitol (250 ml) the day before surgery, at the beginning of the surgery (250 ml), and the day after surgery (125 ml). During surgery, fluid maintenance was set at 3 ml/kg/h of normal saline with an infusion pump. Goal-directed fluid restriction was adopt [14]. Volume expansion were carried out with additional colloid (gelatins or hydroxyethyl starches) boluses (200 ml) only in case of hypovolemia. And hypovolemia were defined as systemic hypotension (MAP < 65 mmHg) with cardiac index(CI) < 2.5 L/min/m^2^ and a SVV > 15%.

### Hemodynamic measurements

The FloTrac/Vigileo system (FloTrac, Edwards Life sciences, Irvine, CA) was used to continuously monitor dynamic markers of fluid responsiveness (cardiac output (CO), CI, stroke volume (SV) and SVV).

Diameter of left ventricular outflow tract (D_LVOT_) was measured by B-mode ultrasonography at the left ventricle long axis plane in the middle of the esophagus after anesthesia induction and before the surgery. The operation procedure is to put the probe into the middle of the esophagus (about 20 cm from the incisors) to show the four-chamber heart view, then keep the probe still, and obtain the two-chamber view by adjusting the angle and direction of the ultrasonic probe. The aortic valve and left ventricular outflow tract can be seen in the long axis direction. In the systolic period, the distance between the left ventricular endometrium and the medial margin of the anterior mitral valve was measured at 1 cm beneath the aortic valve.

Velocity time integral (VTI) of left ventricular outflow tract (VTI_LVOT_) was measured after anesthesia induction and during the surgery. VTI represents the moving distance of erythrocytes in one systolic period, and is computed by spectral Doppler imaging at the long axis plane of the deep stomach. To obtain image of this plane, we should put the probe into the stomach cavity, and adjust the probe to press it against the gastric mucosa until the left ventricular apex is shown on the top of the image. In order to show the left ventricular outflow tract and the aortic valve in the center of the image, it is necessary to bend the probe to the left. The spectral Doppler sampling volume should be placed beneath the aortic valve, to make the direction of the beam parallel to the ventricular septum, and then adjust the probe direction to read the maximum flow rate and then trace the outline by means of color Doppler flow guidance and audio signals as well as spectral form.

During the surgery, VTI_LVOT_ were measured every30 minutes. One well-trained expert collected all of the echocardiographic data. Area of left ventricular end-diastolic volume (LVEDA), LVEDV and LVEDVV was then calculated using the following formulas:

$$ \mathrm{LVEDA}=3.14\times {{\mathrm{D}}_{\mathrm{LVOT}}}^2\div 4 $$

$$ \mathrm{LVEDV}=\mathrm{LVEDA}\times {\mathrm{VTI}}_{\mathrm{LVOT}} $$

$$ \mathrm{LVEDVV}=2\times \frac{{\mathrm{LVEDV}}_{\mathrm{max}}-{\mathrm{LVEDV}}_{\mathrm{min}}}{{\mathrm{LVEDV}}_{\mathrm{max}}+{\mathrm{LVEDV}}_{\mathrm{min}}}\times 100\% $$

where LVEDV_max_ and LVEDV_min_ are the maximal and minimal values within one respiratory cycle. All hemodynamic data were recorded after 3 min of hemodynamic stability.

### Statistical methods

Data collected during the study were compiled using Microsoft Office Excel. Normality of data was tested with Kolmogorov–Smirnov one-sample test. Variates were presented as mean ± SD for continuous variates with normal distribution. And variates with non-normal distribution were presented as median (inter quartile range). The correlation between SVV and LVEDVV was assessed using Pearson correlation coefficient. Bland-Altman analysis was performed to evaluate the agreement between SVV and LVEDVV. Receiver operating characteristic (ROC) curves were generated for SVV and LVEDVV varying the discriminating threshold of each parameter and area under the ROC curves were calculated. All statistical assessments were two-side. A *P* value <0.05 was considered as statistically significant. All statistical analyses were carried out with IBM SPSS Statistics for Windows, version 21.0 (IBM Corp, USA).

## Result

A total of 33 patients with mean age of 64.4 years undergoing craniotomy were enrolled in the present study. Transesophageal echocardiography data of 7 patients were unavailable. Thus, 26 patients (10 males and 16 females) were included in the final analysis. Ten patients received fluid expansion during the surgery and a total of 145 pairs data were obtained. The demographic characteristic and medical conditions of patients are presented in Table 1. 53.8% patients did the surgery for the reason of brain tumor and 46.2% patients for intracranial aneurysm. About 70% of patients had comorbidities of hypertension.

**Table 1:** Baseline of 26 patients

Characteristic	*N* = 26
Age (year)	64.4 ± 9.0
Gender	
Male	10 (38.5%)
Female	16 (61.5%)
ASA III	25 (96.2%)
ASA IV	1 (3.8%)
Weight (kg)	57.5 ± 8.9
Height (cm)	159.9 ± 7.6
BSA (m^2^)	1.6 ± 9.0
Diagnosis	
Intracranial mass	14 (53.8%)
Intracranial aneurysm	12 (46.2%)
Comorbidities	
Hypertension	18 (69.2%)
Diabetes mellitus	3 (11.5%)
COPD	7 (26.9%)

Data were presented as mean ± SD or absolute number (percentage)

BMI: body mass index; BSA: body surface area. COPD: chronic obstructive pulmonary disease

Changes in hemodynamic variables after volume expansion are presented in Table 2. Volume expansion induced a significant increase in CO (from 3.5 ± 0.5 to 4.0 ± 0.6 L·min^−1^·m^−2^, *P* = 0.006) and CI (from 2.2 ± 0.2 to 2.6 ± 0.4, *P* = 0.008). At the same time, we observed significant decreases in both Vigileo-SVV (from 17.8 ± 2.8 to 11.0 ± 2.8%, *P* < 0.001) and LVEDVV (from 22.1 ± 7.3 to 13.6 ± 3.8%, P < 0.001). No significant changes were found in Vigileo-SV (from 54.4 ± 10.0 to 59.3 ± 8.5, *P* = 0.084), VTI (from 18.7 ± 2.7 to 19.5 ± 3.4%, *P* = 0.503) and TEE-SV (from 53.3 ± 8.0 to 58.2 ± 10.7, *P* = 0.156).

**Table 2:** Hemodynamic variables before and after fluid expansion

	Before FE	After FE	*P* value
CO (L•min^−1^)	3.5 ± 0.5	4.0 ± 0.6	0.006*
CI (L•min^−1^•m^−2^)	2.2 ± 0.2	2.6 ± 0.4	0.008*
SV (L)	54.4 ± 9.9	59.3 ± 8.5	0.084
SVV (%)	17.8 ± 2. 8	11.0 ± 2.8	<0.001*
VTI-LVOT (cm)	18.7 ± 2.7	19.5 ± 3.4	0.503
LVEDV (L)	53.3 ± 8.0	58.2 ± 10.7	0.156
LVEDVV (%)	22.1 ± 7.3	13.6 ± 3.8	<0.001*

FE, fluid expansion; CI, cardiac index; CO, cardiac output; SV, stroke volume; SVV, stroke volume variation; VTI-LVOT: velocity time integral of left ventricular outflow tract

*P < 0.05 before FE vs. after FE

Data were presented as mean ± SD

**Fig. 1:**
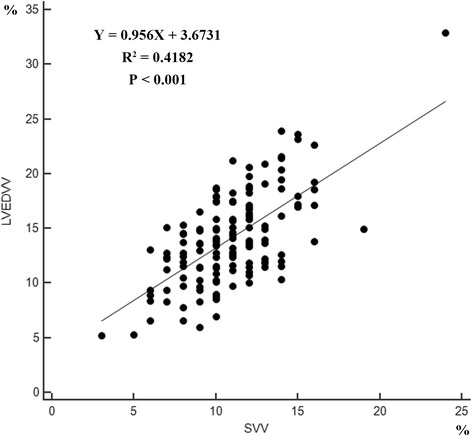
Correlation between the Left ventricular end-diastolic volume variation (LVEDVV) estimated by left ventricular short diameter of axle using TEE and stroke volume variation (SVV) obtained with the FloTrac/Vigileo monitor. (Y = 0.956X + 3.6731, R^2^ = 0.4182, *P* < 0.001)

**Fig. 2:**
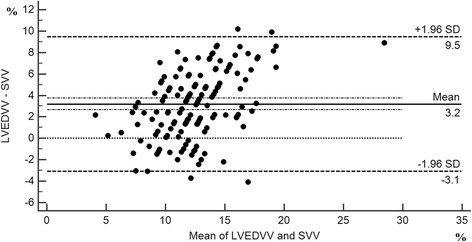
Bland-Altman plot of fluid responsiveness measurement obtained using the transesophageal echocardiography (TEE) in comparison to SVV obtained with the FloTrac/Vigileo monitor. SVV: stroke volume variation from the FloTrac/Vigileo monitor; LVEDVV: Left ventricular end-diastolic volume variation estimated by left ventricular short diameter of axle using TEE. The bias and precision of the two methods were 3.4% and 4.85%%, respectively. (Bias = 3.4% ± 4.9%)

**Fig. 3:**
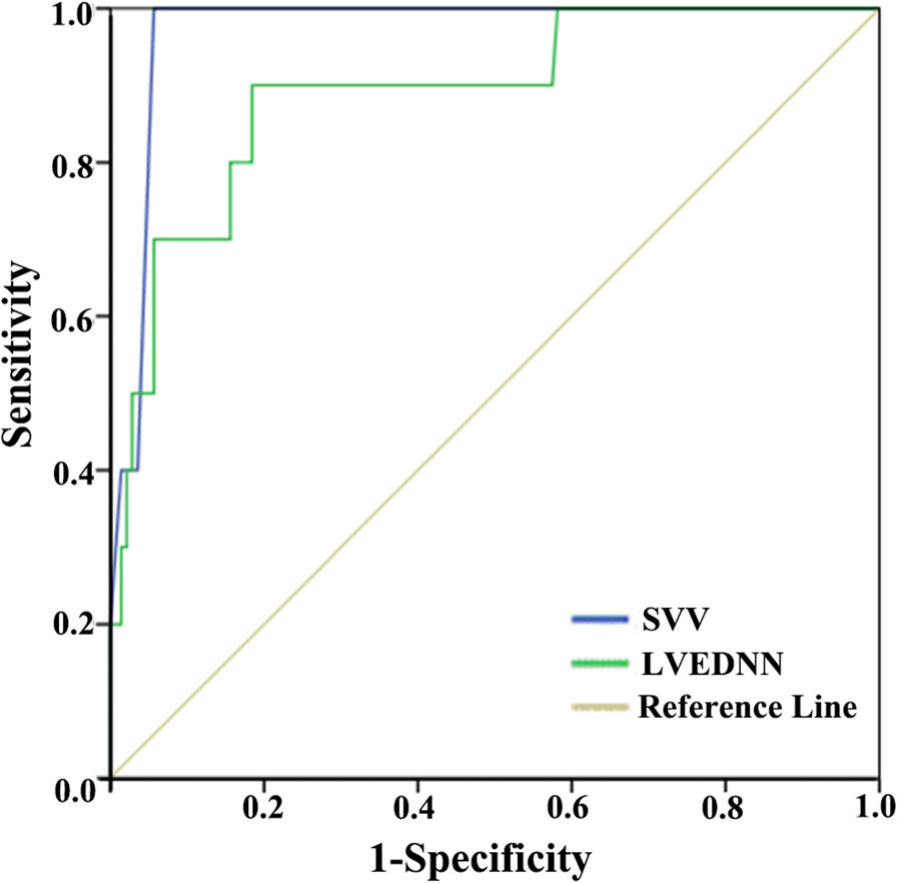
Receiver operating characteristic (ROC) curves comparing the ability of SVV obtain from FloTrac/Vigileo and LVEDVV obtain from TEE to discriminate hypovolemia (systemic hypotension (MAP <65 mmHg) associated with a CI <2.5 L/min/m2 and a SVV > 15%.). Area under the curve is SVV 0.971 (0.945–0.997) (P < 0.001), LVEDVV 0.890 (0.783–0.998) (P < 0.001) respectively

A significant correlation was found between SVV and LVEDVV obtained by TEE (R^2^ = 0.4182, *P* < 0.001) (Fig. 1). Bland-Altman analysis of pooled data is presented in Fig. 2. The mean bias and precisions of LVEDVV and SVV are 3.4% and 4.85%, respectively (Fig. 2). The area under the ROC curve are as follows: 0.971 (95%CI: 0.945–0.997) for SVV (P < 0.001), 0.890 (95%CI: 0.783–0.998) for LVEDVV (P < 0.001) (Fig. 3). The sensitivity of SVV (15%) is 0.990 and the specificity is 0.975. A threshold value of LVEDVV is greater than 15.3% to help discriminate hypovolemia with a sensitivity of 0.912 and a specificity of 0.815.

## Discussion

In this study, we examined the validity of monitoring perioperative fluid volumes in neurosurgical patients using LVEDVV estimated by left ventricular short diameter of axle using TEE (LVEDVV), compared with a golden standard SVV obtained by the FloTrac/Vigileo monitor. Our study results showed that LVEDVV agreed well with the reference measurement SVV.

As we know, it is important to evaluate fluid responsiveness in high-risk patients undergoing brain surgery. Most dynamic indexes are based on respiratory ΔSV and are better than static indexes to predict fluid responsiveness. Although these indexes were obtained by minimally invasive cardiac output monitors, but they still required invasive and specific devices. Such as evaluation of SVV with FloTrac/Vigileo system requires only intra-arterial cannulation which is also a necessity for intracranial mass and aneurysm operations. However, TEE is invasiveness and ease of application. It has been shown that TEE could improve clinical outcome and decrease postoperative morbidity [15–17], which is the primary method to accurately evaluate cardiac function during surgery. LVEDVV is an easily obtained parameter through Esophageal Doppler detection. The results of our study showed that LVEDVV agreed well with the SVV obtain through FloTrac/Vigileo in prediction of fluid responsiveness.

We used SVV obtained from FloTrac/Vigileo monitor as the “gold standard” . This continuous monitoring system (FloTrac, Edwards Life sciences, Irvine, CA) has been tested clinically in many critically ill subjects in cardiac surgeries and intensive care units [18–20]. The reliability of cardiac output measurement obtained by this system was confirmed. It can provide SVV for fluid management, which has proven to enhance surgical safety in the treatment of critically ill patients [21].

This indicator LVEDVV has potential clinical applications for goal-directed intraoperative fluid administration and situations in which volume and cardiac function monitoring during surgery is important. In most clinical settings, the specific devices and algorithms for advanced hemodynamic monitoring are not always available. The TEE technique can quantify not only LVEDVV but also cardiac function for high-risk patient, which is simple, feasible, and cost effective. In our study, we adopt goal-directed fluid restriction strategy, which has been proved to be benefit for high-risk patients undergoing brain surgery [3], [6, 14], and fluid expansion were allowed only in case of systemic hypotension associated with a CI < 2.5 L/min/m^2^ and a SVV > 15%. The results showed that a threshold value of LVEDVV was greater than 15.3% to discriminate hypovolemia, with a high sensitivity (0.912) and specificity (0.815). Moreover, it has a significant correlation with SVV obtained through FloTrac/Vigileo monitor. Thus the LEVDVV obtained through TEE may be sufficiently reliable to be applied in clinical use to predict fluid responsiveness and assess volume status of surgical patients.

Our study has several limitations. First, echocardiographic measurement requires special training. The accuracy of the data can be affected by the proficiency of echocardiographic measurement. However, in this study, all of the echocardiographic data were measured by one expert with at least two years of work experience. Second, in this study, we only focused on patients with general anesthesia. Further studies in patients undergoing non-general anesthesia surgery will be required to validate accuracy of transthoracic echocardiographic measurement, which can be applied more broadly. Third, this study was performed in a single center, so the results may not be extrapolated to other surgical practices.

## Conclusion

For fluid responsiveness of patients during craniotomy in ASA III-IV, LVEDVV measured by left ventricular short diameter of axle using M type echocaidiographic measurement seems an acceptable monitoring indicator. This accessible method has promising clinical applications in situations where volume and cardiac function monitoring is of great importance during surgery.

## Abbreviations

CI: Cardiac index
CO: Cardiac output
D_LVOT_: Diameter of left ventricular outflow tract
IRB: Institution review board
LVEDA: Area of left ventricular end-diastolic volume
LVEDV: Left ventricular end-diastolic volume
LVEDVV: Left ventricular end-diastolic volume variations
PEEP: Positive end expiratory pressure
SV: Stroke volume
SVV: Stroke volume variation
TEE: Transesophageal echocardiography
VTI: Velocity time integral
VTI_LVOT_: Velocity time integral (VTI) of left ventricular outflow tract
